# Design and development of a gait training system for Parkinson’s disease

**DOI:** 10.1371/journal.pone.0207136

**Published:** 2018-11-12

**Authors:** Ainara Garzo, Paula Alexandra Silva, Nestor Garay-Vitoria, Erik Hernandez, Stephen Cullen, Valérie Cochen De Cock, Petra Ihalainen, Rudi Villing

**Affiliations:** 1 Neuroingeneering Area, Health Division, TECNALIA, San Sebastian, Gipuzkoa, Spain; 2 Department of Design Innovation, Maynooth University, Maynooth, Co. Kildare, Ireland; 3 Computer Architecture and Technology Department, University of the Basque Country (UPV-EHU), San Sebastian, Gipuzkoa, Spain; 4 Clinical Investigation Center, Montpellier University Hospital, Montpellier, France; 5 EuroMov, University of Montpellier, Montpellier, France; 6 Neurological Unit, Beau Soleil Clinic, Montpellier, France; 7 Department of Electronic Engineering, Maynooth University, Maynooth, Co. Kildare, Ireland; Fondazione Ugo Bordoni, ITALY

## Abstract

**Background:**

Rhythmic Auditory Stimulation (RAS) is an effective technique to improve gait and reduce freezing episodes for Persons with Parkinson’s Disease (PwPD). The BeatHealth system, which comprises a mobile application, gait sensors, and a website, exploits the potential of the RAS technique. This paper describes the tools used for co-designing and evaluating the system and discusses the results and conclusions.

**Methods:**

Personas, interviews, use cases, and ethnographic observations were used to define the functional requirements of the system. Low fidelity prototypes were created for iterative and incremental evaluation with end-users. Field trials were also performed with the final system. The process followed a user centered design methodology defined for this project with the aim of building a useful, usable, and easy-to-use system.

**Results:**

Functional requirements of the system were produced as a result of the initial exploration phase. Building upon these, mock-ups for the BeatHealth system were created. The mobile application was iterated twice, with the second version of it achieving a rating of 75 when assessed by participants through the System Usability Scale (SUS). After another iteration field trials were performed and the mobile application was rated with an average 78.6 using SUS. Participants rated two website mock-ups, one for health professionals and another for end-users, as good except from minor issues related to visual design (e.g. font size), which were resolved in the final version.

**Conclusion:**

The high ratings obtained in the evaluation of the BeatHealth system demonstrate the benefit of applying a user centered design methodology which involves stakeholders from the very beginning. Other important lessons were learned through the process of design and development of the system, such as the importance of motivational aspects, the techniques which work best, and the extra care that has to be taken when evaluating non-functional mock-ups with end users.

## Introduction

External rhythmic cues can be used with success to rehabilitate motor functions in patients with movement disorders [1]. An example from clinical neurology is provided by gait disorders in idiopathic Parkinson’s Disease (PD). The main symptoms of PD (i.e., resting tremor, bradykinesia, and postural instability) are typically associated with gait disorders (i.e., reduced stride length and walking speed, freezing periods), which often lead to falls [2–6]. Rhythmic cueing is applied in order to provide a reference “*healthy movement pattern*” for an individual suffering from gait disorders [7]. When applying Rhythmic Auditory Stimulation (RAS), the patient is asked to match her/his walking speed to a regular stimulus in the form of a repeated isochronous sound (metronome) [8–10] or a piece of music characterized by a salient beat, sometimes with an embedded metronome [11, 12]. RAS is one of the earliest and most popular forms of Neurological Music Therapy [13], and provides a promising therapy for gait impairments in PD [14, 15]. Auditory cues, in comparison to other cueing methods (such as tactile or visual) exhibit the strongest beneficial effects for gait training [7, 16]. In the BeatHealth project a system has been developed to deliver a training session for Persons with PD (PwPD) by exploiting RAS in the form of musical stimuli. The system is formed by:

1. Two sensors, worn on the leg shanks, for recording a user’s gait kinematic data.
2. A mobile application running on a smartphone which receives the information from the sensors and is capable of adapting the music rhythm and synchronizing it with the user’s gait.
3. A cloud platform assessed through a website where relevant information associated with and data generated by the application is safely stored and can be checked by the user and shared with health professionals.

The latter two components both involve a significant design effort:

1. A website designed for health professionals to define the training program to be performed by the PwPD and later to track their progress through the program.
2. A mobile phone application designed for PwPD which loads the training program to be performed by the PwPD that has been designed by the health professional.
3. A website for PwPD to access their own progress data.

This paper explores the following research questions:

- Q1: Has the user centred design (UCD) methodology identified the appropriate functional requirements of the BeatHealth system?
- Q2: How usable was the resulting BeatHealth system?

The design process for the components of the BeatHealth system was iterative and followed a user centered design methodology which actively sought and incorporated the input of health professionals and end-users (both PwPD and caregivers). This paper presents the tools used in this process as well and the results and conclusions of such process.

## Methods

**Fig 1:**
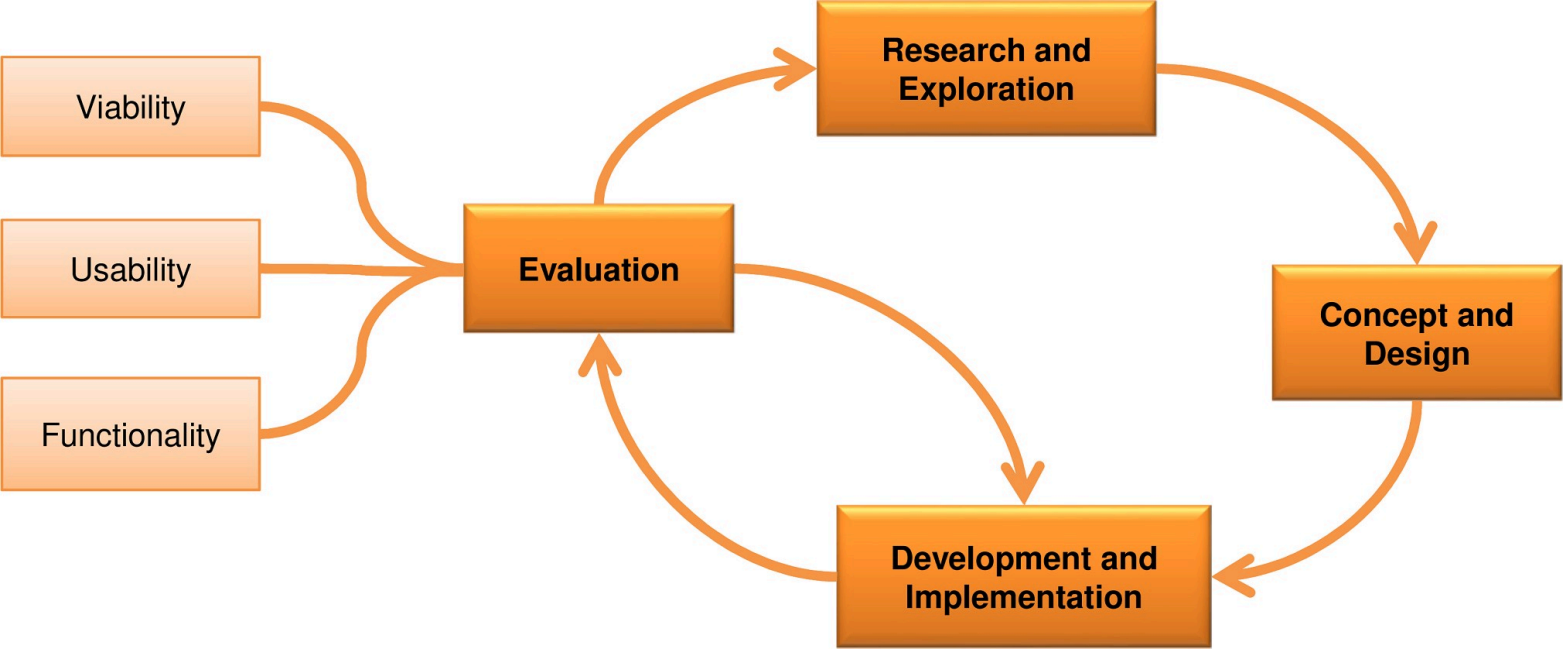
Methodology used in this research.

To build the BeatHealth system, different stakeholders were involved across the various phases of the project with the aim of designing an easy-to-use, easy-to-learn, and usable system. The methodology used in this project is iterative and includes elements from several approaches such as the ISTAG Experience and Application Research (E&AR) framework [17], Design Thinking [18], and User Centered Design (UCD)—ISO 9241–210:2010 [19]. Fig 1 presents an overview of the methodology including the iteration flow between the four phases used during this project.

The four phases of the methodology and its main goals are as follows:

1. Research and exploration: understand the user and his/her behavior, in order to specify the requirements, goals, and context of use.
2. Concept and design: develop early designs to support in the co-design loop with end-users and stakeholders and to fulfill the requirements defined in the previous phase.
3. Development and implementation: iterative development and evaluation by users of the designs created in the previous phase and of the prototypes to be used in next phase.
4. Evaluation: evaluate prototypes with final users to verify that requirements are met and to assess the user interfaces developed in terms of usefulness, usability, acceptance, satisfaction, etc. Depending on the results it may be necessary to start again from phase one and redefine the user requirements or to iterate from phase three by developing a refined prototype. When assessing the system three attributes should be considered: Viability to assess if the prototype fulfills users’ needs and context requirements; Usability to check if the prototype is easy-to-use, is acceptable, and is useful; and Functionality to verify if the prototype is working properly and as expected (from a technical perspective).

In this project, a number of different tools were selected for use in different phases. Ad Hoc Personas [20, 21], Use Case definitions [22], interviews, and ethnographic observations [23] were chosen for the *Research and Exploration* phase. In the *Concept and Design* phase, card sorting tools [24] and Paper Prototyping [25] were used with the aim of building initial low fidelity prototypes. For the *Development and Implementation* and *Evaluation* phases, Wizard-of-Oz [26], Thinking-aloud [27], standard validated questionnaires, semi-functional mock-ups, and interviews were used for building prototypes and evaluating them with final users. The following sections present the results and conclusions of this process, including the perception of the participants who assessed the usability of the system.

During the BeatHealth project, PwPD, relatives, and health professionals were involved in the design of the application and whole system. In the final phases, end-users also evaluated the system in their own homes for three months.

### Design and development process

#### Research and exploration phase

At the beginning of this project, a number of tools were used to gain a better understanding of the usage context and the stakeholders. Initially, Ad Hoc Personas were defined based on the experience and knowledge of the multidisciplinary team that participated in the process. Those Personas were then used to better understand the different final user profiles and context of use. After defining Personas, with the aim of identifying the main functionality required by the system, semi-structured interviews, use case definitions, and ethnographic observations were used as explained in the following sections.

#### Interviews

Twelve PwPD and two relatives of PwPD were interviewed about the utility of the BeatHealth system and its main functionalities. The interview participant profiles were heterogeneous in terms of disease severity and medication for both PwPD and relatives. An interview guide was prepared in advance and adjusted depending on the flow of the interview and the information provided by the participants.

As result of the interviews, ten PwPD said that they prefer to use this kind of system by themselves rather than under clinical supervision. The other two PwPD did not show any interest in the application. During the interview, PwPD were asked about what relevant information the application could provide for them. They did not show particular interest in accessing data regarding their health status. However, when they were asked about their desired information from training sessions they indicated a preference for having access to step length, number of steps, and time of training. The main concern of PwPD was the type of headphones they could use with the system because they preferred to use larger headphones that were easier to manage with dexterity impairments. The relatives who were interviewed did not show interest in having access to PwPD’s data, either to monitor the usage of the system or the patient’s progress.

#### Use case definitions

Use Cases were used to identify the main behavioral functionality of the system. This tool was very useful to differentiate between general functionalities of the system (such as “log-in”, “log-out” or “play music”) and more specific ones (such as “start a new training session” or “see my data”). Use case definitions proved to be very useful for multidisciplinary work and allowed researchers with different profiles to participate in the definition. These included health professionals, technicians, engineers, sports professionals, designers and developers. In S1 Table can be found the template used for use case definition.

The information collected using this template was shared among all participants. In order to indicate the relevance of each Use Case, the MoSCoW scale [28] was used. Every participant was asked to rate the importance of each Use Case.

As a result of using this tool the most relevant functionalities were selected for implementation in the final system. These were included in the functional requirements of the system used by the technical members of the project team and the remaining (less highly rated) functionalities were noted as desirable or optional for future application improvements. Following section summarizes results obtained in the exploration phase.

#### Ethnographic observations

Ethnographic observation was the method used to gather data about the potential users of the proof-of-concept product and the effect that the symptoms of PD may have on their interactions with their environment in various contexts. This method was used to inform the design of the prototypes and the plan for the design process itself.

Several observation sessions were conducted: two with PwPD and one with a healthy participant. Each session was intentionally designed around different contexts to gather understanding of a broader range of movement evaluation tools and techniques and their impact on participants.

The first observation session was conducted by the research team at Centre Hospitalier Régional Universitaire de Montpellier (CHRU). One PwPD participated and was asked to carry out various tasks while wearing headphones to deliver varying rhythmic stimuli and monitoring equipment designed to capture movement and heart rate data. The tasks mainly consisted of walking around a circuit marked out by orange cones while listening to music with varying rhythmic characteristics.

Fitting the equipment took approximately 15 minutes due to the number and nature of components required. No obvious signs of frustration were observed.

It was noted that this prototype required at least one person experienced in fitting the equipment together with one other person assisting, in addition to the participant. The time period taken to fit the equipment and the awkward nature of the process (the participant had to move in various ways to accommodate the fitting of equipment, including raising arms, raising feet, holding arms still and standing still) gave an opportunity for interaction between the participant and the researchers. This was an opportunity for the researchers to develop a relationship with the participant and gain insights to the disposition of the participant prior to the actual testing.

The most significant observation made at this stage was that of an increased tremor when the participant was trying to physically stay still or focusing on a mechanical task. Studies into motivation, reward, and PD [29] show that apathy may be observed in a PwPD. What was observed in this trial was an interest and curiosity by the participant into the equipment used and the overall study. There was also an evident enthusiasm and determination in the participant to complete the trial tasks.

The second session was conducted by the CHRU team with a healthy participant in order to compare the results with the first observation session with a PwPD. The main observations of the researcher were that 1) the participant appeared to be more relaxed, 2) seemed less interested, 3) asked fewer questions about the evaluations, and 4) focused less on the fitting of the equipment.

The setting for the third session was an office environment, and featured a PwPD attending a medical consultant at the Clinique Beau-Soleil. During this consultation, the participant was asked to complete some concrete tasks using a memory app on a smart phone to evaluate general issues related to managing a touch screen. Several studies show the effects of PD symptoms on the ability to multitask and that working memory tasks interfere with the gait of PwPD [30]. Therefore, the participant was asked to perform tasks in different situations that were similar to the BeatHealth application scenario. It was observed that there was no difficulty performing the memory test while sitting down. When the participant was asked to complete the test while standing he was able to do so. When the participant was asked to complete the same test while walking the participant froze just after beginning and was not able to complete either task, that of walking or the memory task.

Witnessing this participant’s experience gave the observer a deeper understanding of user needs and the effects of PD symptoms on the user’s interaction with their environment.

This ultimately helped to inform the design and function of the final Proof of Concept (PoC) and the intermediate prototypes and evaluation techniques used in the process.

Below the lessons learned from the Ethnographic Observations are presented as result of the work done.

The use of ethnographic observations as an approach to understand the potential users of the future final product provided valuable insights into how PwPD may feel during evaluations. It confirmed the value of existing proposed methods and inspired thought into what may improve the methods being planned for the PoC user-interface evaluations.

In short, using ethnographic observations in this project led to the following:

- Breaking down of the evaluation process into small sections of shorter duration and allowing a break between sections. This also helped to minimize the monotony of any of the repetitiveness of the evaluations.
- Reducing moments of multitasking, such as, for example, delivering feedback while a task is ongoing.
- Highlighting the importance of including elements of motivation and goal setting.
- Reducing the need for participants to enter data into the app.
- Simplifying the set-up and placement of PoC equipment.
- Creating clear and simple instructions.
- Clearly communicating that the participant is contributing to a shared goal.
- Designing the user interface and the tasks to test it for the ability range of PwPD.
- Specifying the appropriate distance to be walked during the task.
- Designing the PoC study tasks to be composed of smaller achievable elements and clearly identifying when these elements are completed. Without this, the determination of individual participants to complete tasks could put them at risk.

#### Results of exploration phase: Functional requirements

As a result of the exploration phase, a set of preliminary functional requirements were defined based on the input observations, interviews, and technical expert contributions. The list of functional requirements for the mobile application can be found in S1 File.

Based on the Use Case definitions, Interviews and Ethnographic Observations it was decided to implement a web application whose functionality would simplify the mobile phone application and add value to the whole system. The requirements indicated that there should be two different websites depending on the type of user: end-user (PwPD) or health professional (physician, neurologist, or similar). The website for health professionals should give the user the opportunity to create a training program for the PwPD and track his/her progress. To maintain privacy, the PwPD must give authorization for this feature to his/her physician in advance. A PwPD will be able to directly access their own recorded data using their credentials via the end-user website. Functional requirements for the end-user (PwPD) website and for the health professionals website can be found in S2 and S3 Files respectively.

**Fig 2:**
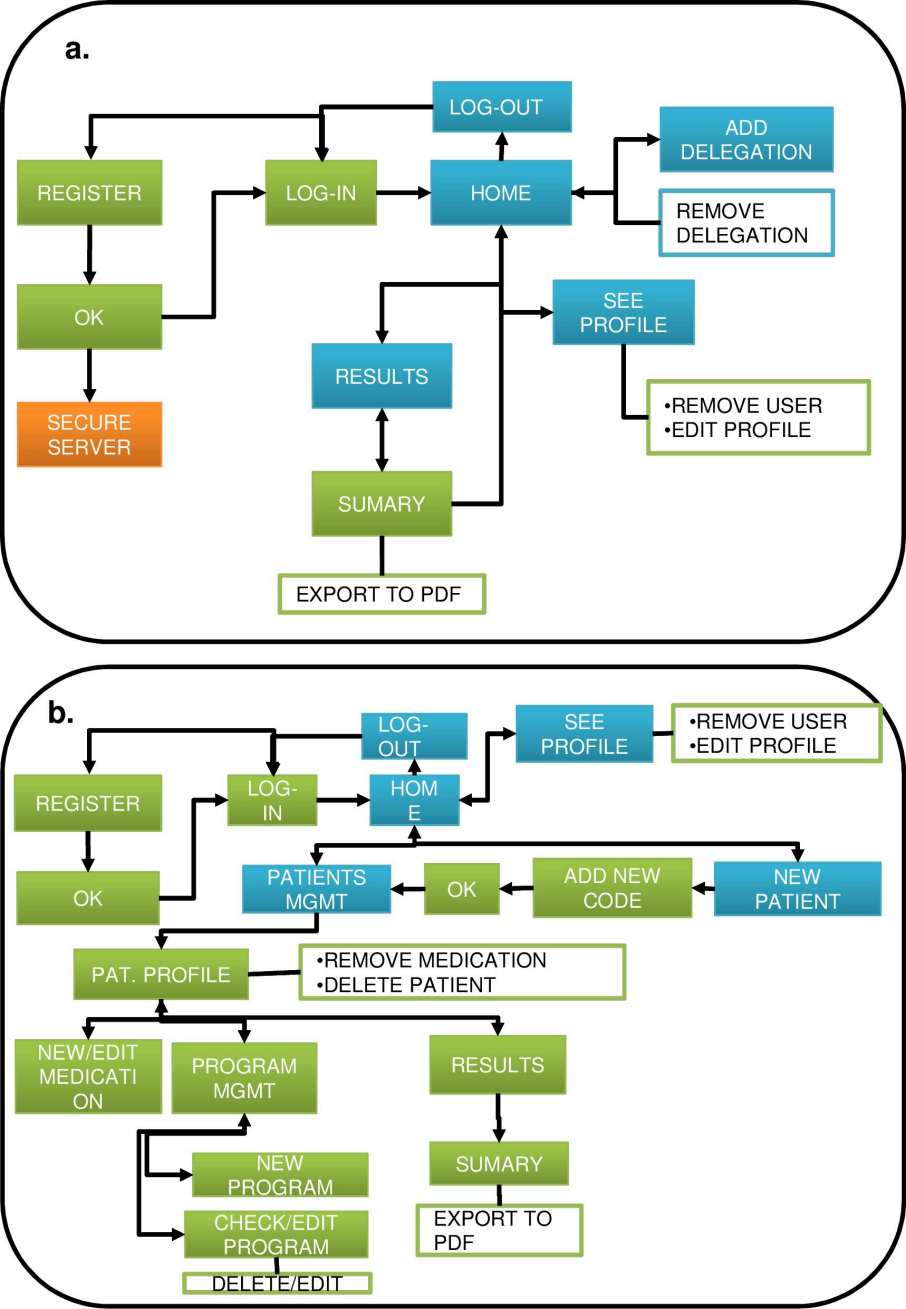
Website flow diagrams. The websites’ main screens are represented by blue squares. Secondary screens are represented with green. Possible actions that do not imply a new screen are represented by white squares. The secure server, which is an external server, is represented with orange because it will be implemented separately from the website for security reasons. The flow among actions and screens are represented with arrows. (a) End-user website flow chart. (b) Health professional website flow chart.

Given these requirements, a preliminary definition of the main system functionalities and actions was defined for the websites. Fig 2 provides an overview of the flow between screens.

**Fig 3:**
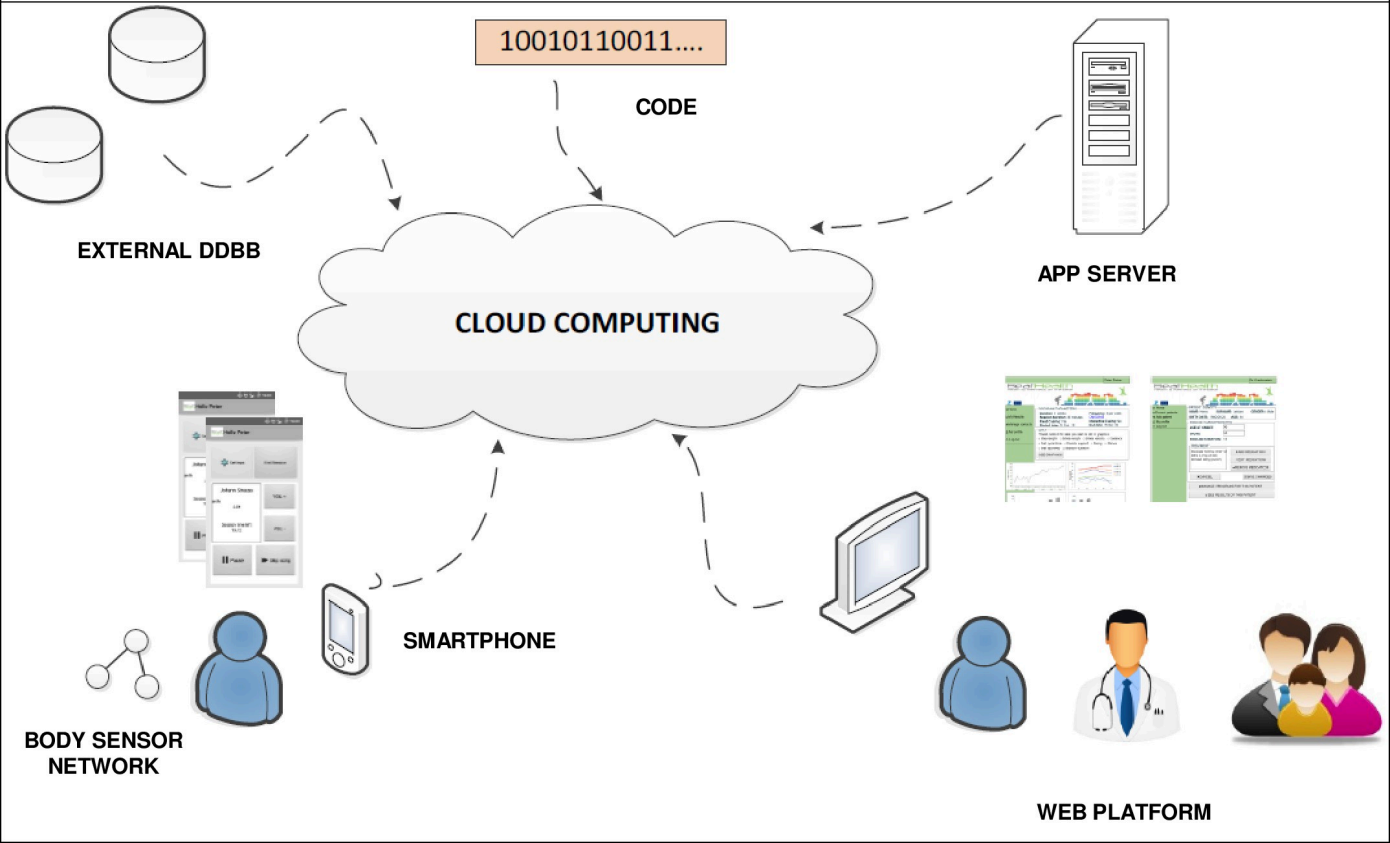
BeatHealth global architecture components. PwPD will use BeatHealth system from the smartphone app which records their data and also allows them to access a brief summary of their own data. PwPD can access to their complete data saved in the cloud from the end user website. Health professionals can monitor PwPD data using the health professional website if they have been previously granted access by the PwPD. Data is saved in an external database and processed by cloud computing. The saved data requires a high level of security and access control.

Both websites need to access user data and present this data in the form of charts, tables, etc. Extensive data should be saved and must be accessible from different places depending on the final users (health professionals and PwPD). Therefore, in BeatHealth this data was stored in the cloud to make access possible for final users everywhere. Cloud services were leased from commercial providers. Fig 3 gives an overview of the global architecture components of BeatHealth.

Due to the sensitive nature of the data, the level of security and access control required was very restrictive and a secure backend was designed to comply the European Directive on Personal Data Protection and the laws of Spain [31, 32] and France [33, 34] where the BeatHealth system was to be piloted [35].

### Concept and design phase

After defining the main functionalities of the system in the research and exploration phase, a number of low fidelity prototypes [36] were built. The goal of these prototypes was to implement all the required functionalities to be tested with stakeholders, including both health professionals who are experts in PD and some PwPD with the aim of identifying any design issues.

**Fig 4:**
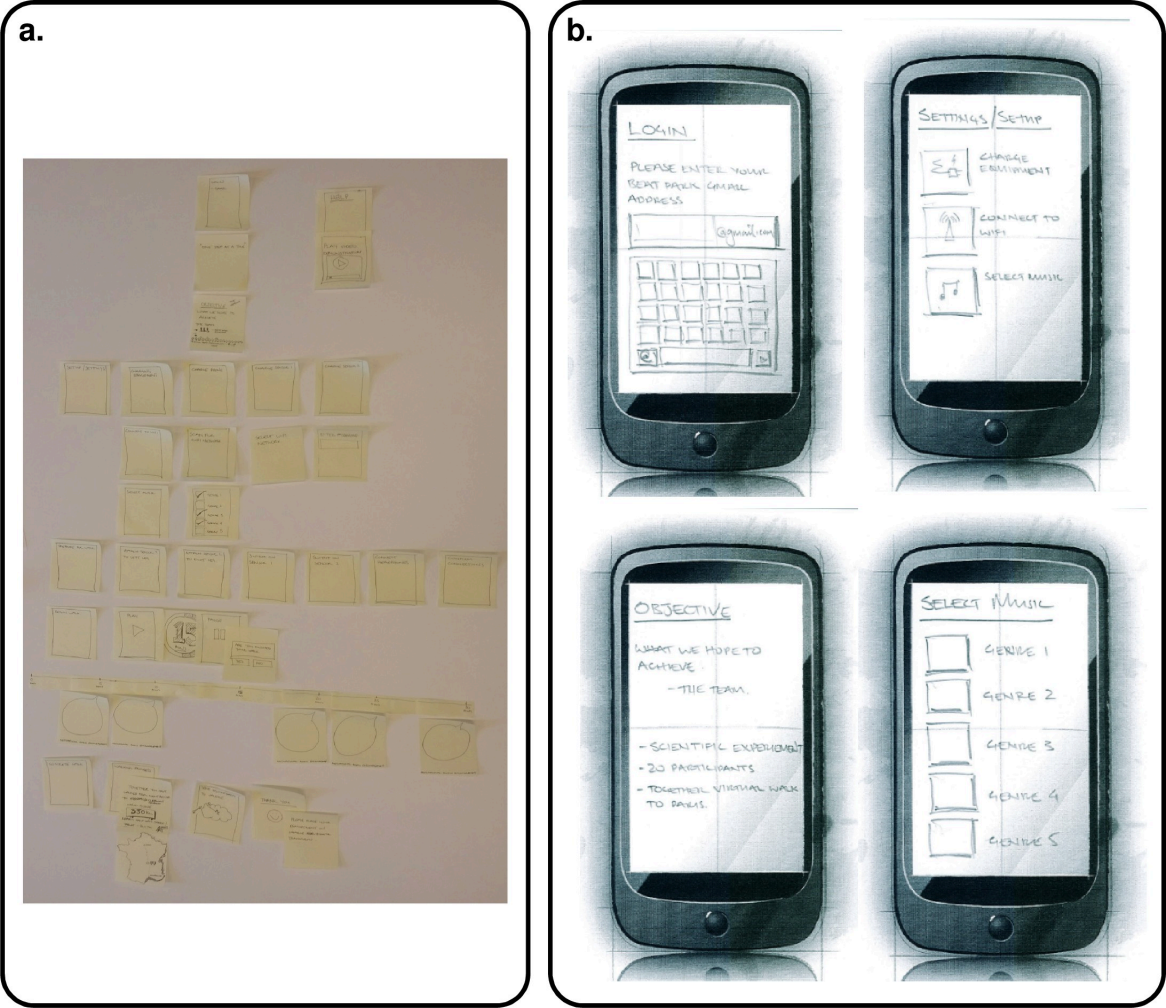
Concept and design tools used. (a) Card sorting example. (b) Paper prototyping example.

The specific low fidelity prototype technique used for co-design with users was paper prototyping as shown in Fig 4. All the paper prototypes generated in this project were designed using the Sqetch toolkit [37]. Card sorting was used to best arrange the required interaction flow. Simple cards containing just screen names were arranged and rearranged to understand the various options and to help decide on the most appropriate workflow.

#### Paper prototyping for co-design

**Fig 5:**
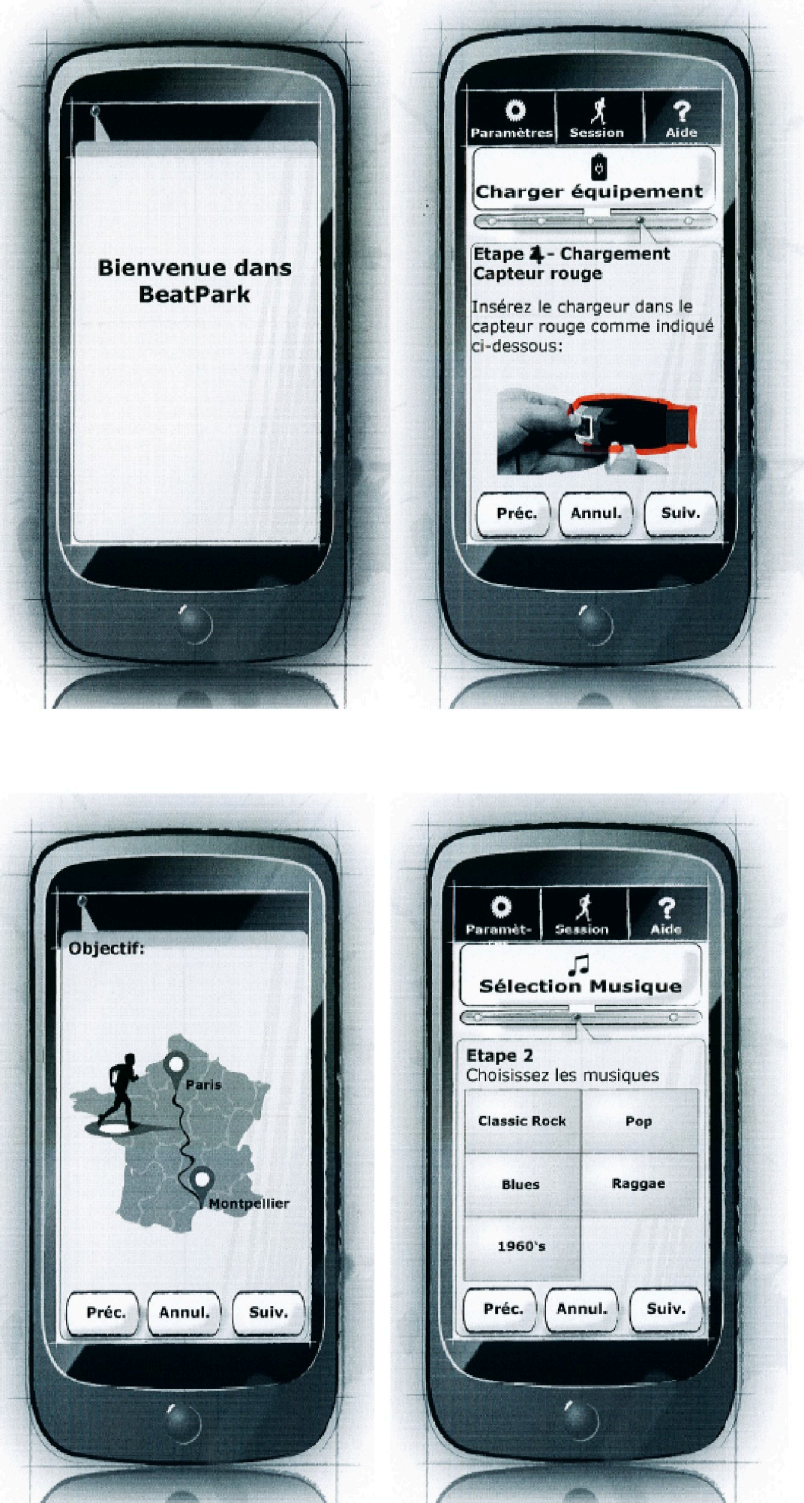
Example of the mock-up used in the participatory design.

An initial application mock-up using paper prototyping was evaluated by experts and stakeholders but no PwPD. Based on the review of this initial prototype, a refined second mock-up was created. Fig 5 shows the second mock-up, which was provided as reference to help participants envision how the technology would look and function [38].

Evaluation meetings were scheduled at the Clinique Beau-Soleil and at CHRU by a multidisciplinary team including: one neurologist, one consultant, one physiotherapist, one PwPD, one human-computer interaction specialist, one computer science specialist, and one computer interaction designer specialized in accessible design.

#### Results of the concept and design phase

**Fig 6:**
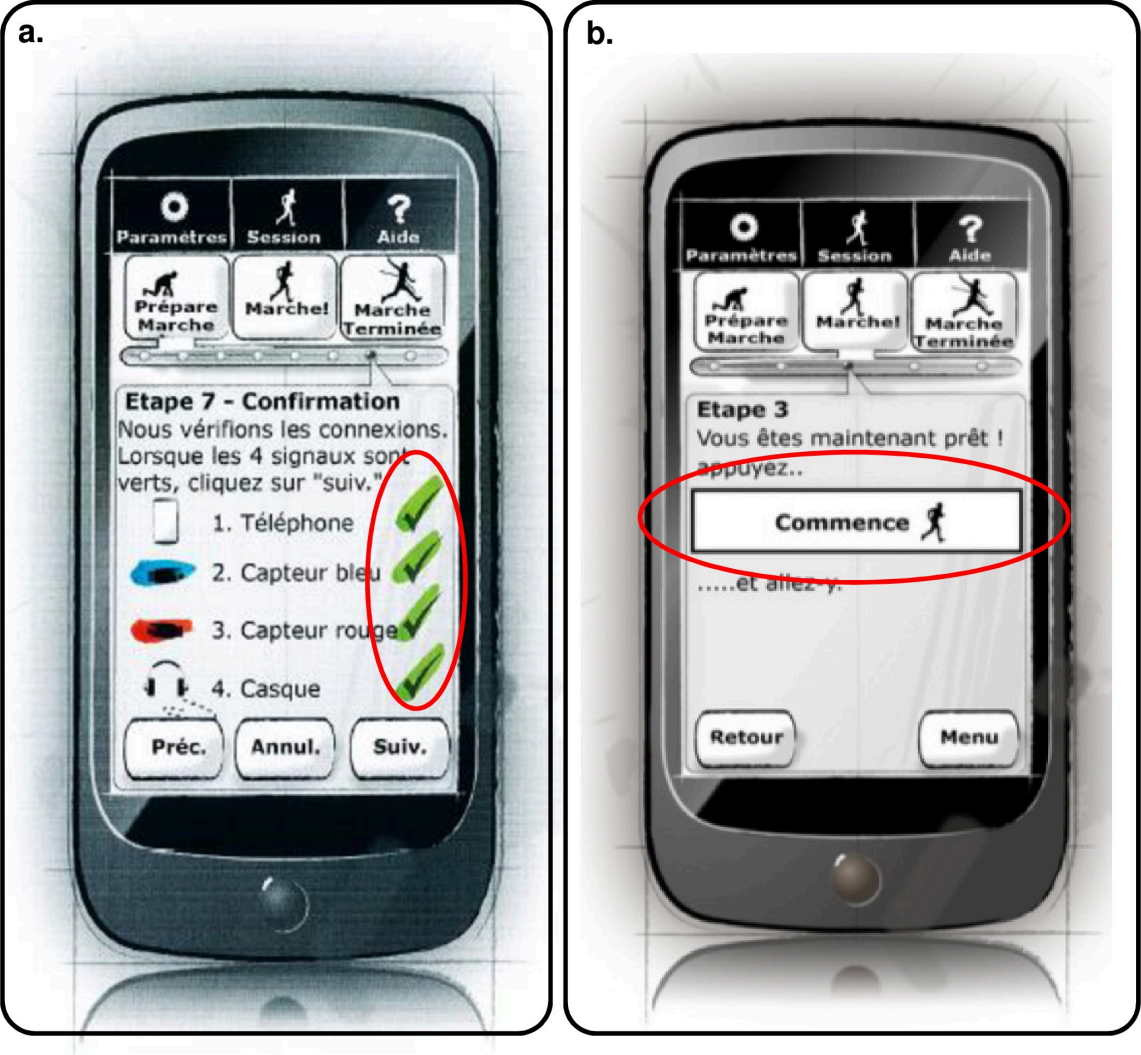
Screen shots. (a) Shows an example of tick symbol considered to be changed. (b) Includes play button in the mock-up used in participatory design.

The main outcomes of this first evaluation were as follows:

1. The workflow required review. In the mobile phone application data should be sent via Wi-Fi connection to the cloud and this required user action. To encourage the participant to send the data, the progress screens should be located after the data sending screens. It should not be possible to begin a new session or to see the progress screens without sending the data first.
2. The sequence for preparing the sensors required change because configuring the sensors in every session was annoying.
3. The login screen might be reduced to a password for a specific design for trials (although perhaps not for a commercial application), reducing the information input and simplifying the process for participants.
4. Support for an AZERTY keyboard (French version keyboard) should be added to cater for varying ability and preference because the final trial users would be French.
5. Abbreviated words needed to be replaced by complete words because some of them were misunderstood.
6. Several words needed to be clarified, e.g., Suite & Suiv.
7. The map screen needed improvement.
8. The layout of the Next and Back buttons required change.
9. The phone charging image needed to be improved because it was not clear enough.
10. The volume buttons needed to be modified to be more useful.
11. The Tick symbol required change because it was not considered to be universal (Fig 6A).
12. The play and pause icons required modification to capture user attention. Fig 6B. shows the the button appearance in the design that had been evaluated.
13. Text spacing between words and lines needed to be modified for improving reading.

Based on these results, a third version of the mock-up was created (referred to as the mobile application first iteration in the next section) and subjected to user experience evaluation in the following phase.

### Development & implementation and evaluation phases

Based on the feedback gathered in the exploration and design phases (“Results of Exploration Phase” and “Results of the Concept and Design Phase” sections) revised designs were implemented for the mobile application and web applications. Over the course of the development and evaluation phase, two iterations of the mobile-phone application design were evaluated and one iteration of the web applications was assessed by PwPD. Evaluating the viability of the whole system including the current design of the user interface was a key objective of this phase of the work.

The mobile application design applied guidelines related to button size, space between buttons, letter size, and contrast for mobile applications targeted at older persons and PwPD [39–41]. Some of the design guidelines (relating to text and letter size or contrast) were also applied to the web application. In addition, scrolling was avoided as much as possible as recommended for PwPD [42].

#### Mobile application evaluation—First iteration

**Fig 7:**
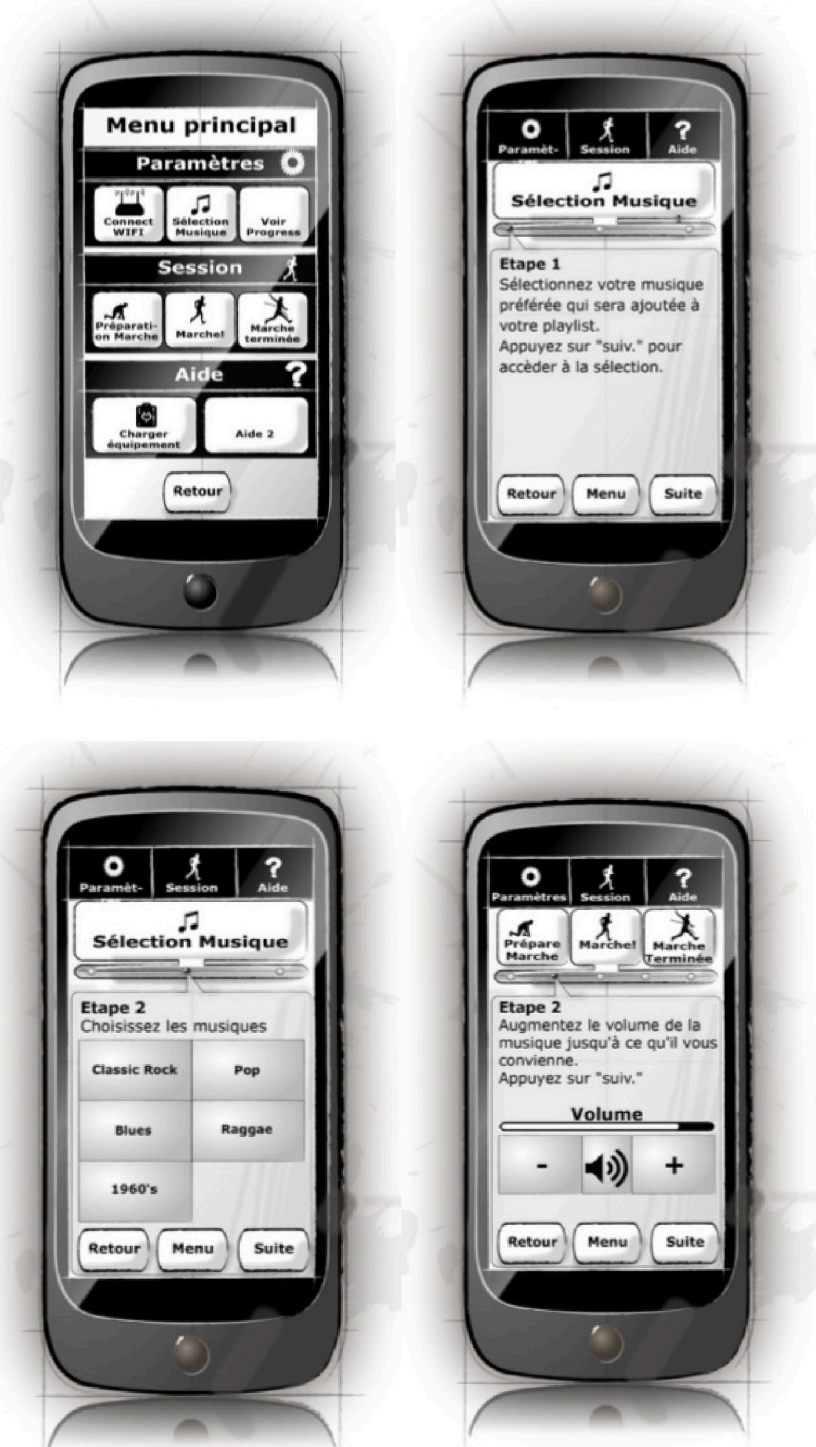
Paper prototype screens used in the Wizard-of-Oz mobile application mock-up evaluated in the first iteration.

The mobile application was evaluated using the Wizard-of-Oz method that enabled user interactions with physical system components and prototype screens. Users attempted to progress through a screen-by-screen task flow using the prototype screens where additional tasks included putting on sensors, connecting the charger, connecting headphones, and connecting to the WiFi network. The think-aloud method was used to gain insight into the user’s thought processes and any difficulties they encountered. The evaluations took place at the Beau Soleil clinic and were conducted by two researchers: one acting as facilitator, helping the user, and the other taking notes. The prototype screens being evaluated were presented to users as a low fidelity paper prototype on A4 pages (see example screens in Fig 7). The prototype screens were in French because the participants were French speakers.

Four PwPD (one female, three males, 64–74 years old, mean age 67.5 years old) participated in the evaluation of the first mobile application iteration. All participants were over 18 years old and gave informed consent to their participation. In addition, all participants belonged to a social security scheme, or were affiliated with such a regime, according to the French law addressing clinical trials involving medical devices (art. L1121-11 in CSP [43]). Complementary (specific) inclusion criteria for PD were that participants should respond to the diagnosis of idiopathic PD according to the criteria for cooperation in assessments, inclusion, and exclusion established by the United Kingdom PD Society Brain Bank [44, 45]. Furthermore, they should present a walking disorder objectified by the neurologist and they should be able to walk for 30 minutes without technical assistance (e.g. cane or walker).

Participants were excluded if they presented a hearing impairment, another neurological disorder that affected gait, gait disorder that did not result from PD (e.g. orthopedic, rheumatologic), or a severe heart or respiratory failure or balance problem that contra-indicated intensive walking. An additional PD-specific exclusion criterion was that participants must not present evidence of an atypical parkinsonian syndrome (including damaged oculomotor system, early falls, hallucinations, Montreal Cognitive Assessment < 21, or severe early dysautonomia).

Concurrent notes were taken based on observations, questions raised, errors, areas of confusion, and/or recommendations made during the evaluation. The key data recorded for each task included the task success (arriving at the end of the screen-flow for that particular task), the time to perform the task, the action flow within each task in relation to the expected flow, and any areas of confusion or errors. Post evaluation interviews and discussions with participants queried their thoughts about the system and the evaluation experience. The key topics covered in this discussion included the overall impression, any task or steps that may have caused confusion or errors, starting the application, placing the equipment on the body, charging the phone and sensors, connecting to WiFi, and any specific recommendations. The estimated duration of a participant’s evaluation session was 60 minutes, however the actual time varied among participants.

The mobile application was evaluated with nine tasks in this first iteration, which are described in next section. The metrics for evaluation were based on: 1) Task success; 2) Timing; 3) Error/confusion/difficulty; 4) Subjective satisfaction; 5) Task ratings.

A post-task System Usability Scale (SUS) [46–48] validated questionnaire was used to measure the application’s usability.

The results of the first iteration of the evaluation are discussed next. Table 1 shows the tasks that were evaluated and the main results. The results showed that tasks number 3, 5 and 7 were more difficult than other tasks for the participants in terms of time, the need for guidance and error rate. Guidance was also needed for task number 2 while task number 9 exhibited a relatively high error rate. The screens used to communicate objectives to the user and to select music were both completed successfully by all participants with 0% error rate.

**Table 1:** Task evaluation results for the mobile application–First iteration.

Tasks		Completion Score				Completion score	Error rate	Average time on tasks
		P1	P2	P3	P4			
1	Turn on the phone	1	1	1	4	75%	31%	0:34
2	Open the application	1	2	1	4	75%	43%	0:56
3	Log In	1	2	2	2	100%	63%	3:11
4	Objectives	1	1	1	1	100%	0%	0:45
5	Connect to WiFi	4	4	4	4	0%	75%	>1:00
6	Select Music	2	1	1	2	100%	0%	1:14
7	Prepare for walk	3	1	4	3	25%	56%	6:58
8	Walk	2	1	1	2	100%	13%	2:43
9	Complete Walk	2	2	1	1	100%	38%	1:40

A rating score of 1 to 4 was used to evaluate tasks for each participant: 1-Completed; 2-Completed with little guidance; 3-Completed with substantial guidance; 4-Incomplete. Error rate is the weighted error rate due to usability issues with the application. Completion rate is the percentage of participants that completed the task with little or no guidance.

One negative result is that none of the participants succeeded in connecting to WiFi (task number 5) using the standard Android user interface. This task included typing a user name and password for connecting to the WiFi using the Android OS on-screen keyboard and this may be difficult for participants. Therefore, improving the user experience in relation to this task was an important aspect that needed to be addressed in the final version of the mobile application and also has relevance for other mobile applications designed for PwPD.

An SUS evaluation [46, 47] was performed to assess the quality of the interaction of this first iteration of the mobile application, and a rating of 59 was obtained. A key factor that was identified as a contributing to this low score was the length of time the selected tasks took for each participant. This version of the application had many steps (to aid novice users) and one way to reduce the time required to perform tasks is to include shortcuts that allow the user to skip sections or steps with which they are familiar. Participants graded only task number 6 (select music) as very satisfactory. In contrast, task number 5 (connect to WiFi) was graded as very unsatisfactory. The motivational and social element of the application (embedded in tasks number 4 and number 9) were considered a motivational factor by participants.

#### Mobile application evaluation—Second iteration

**Fig 8:**
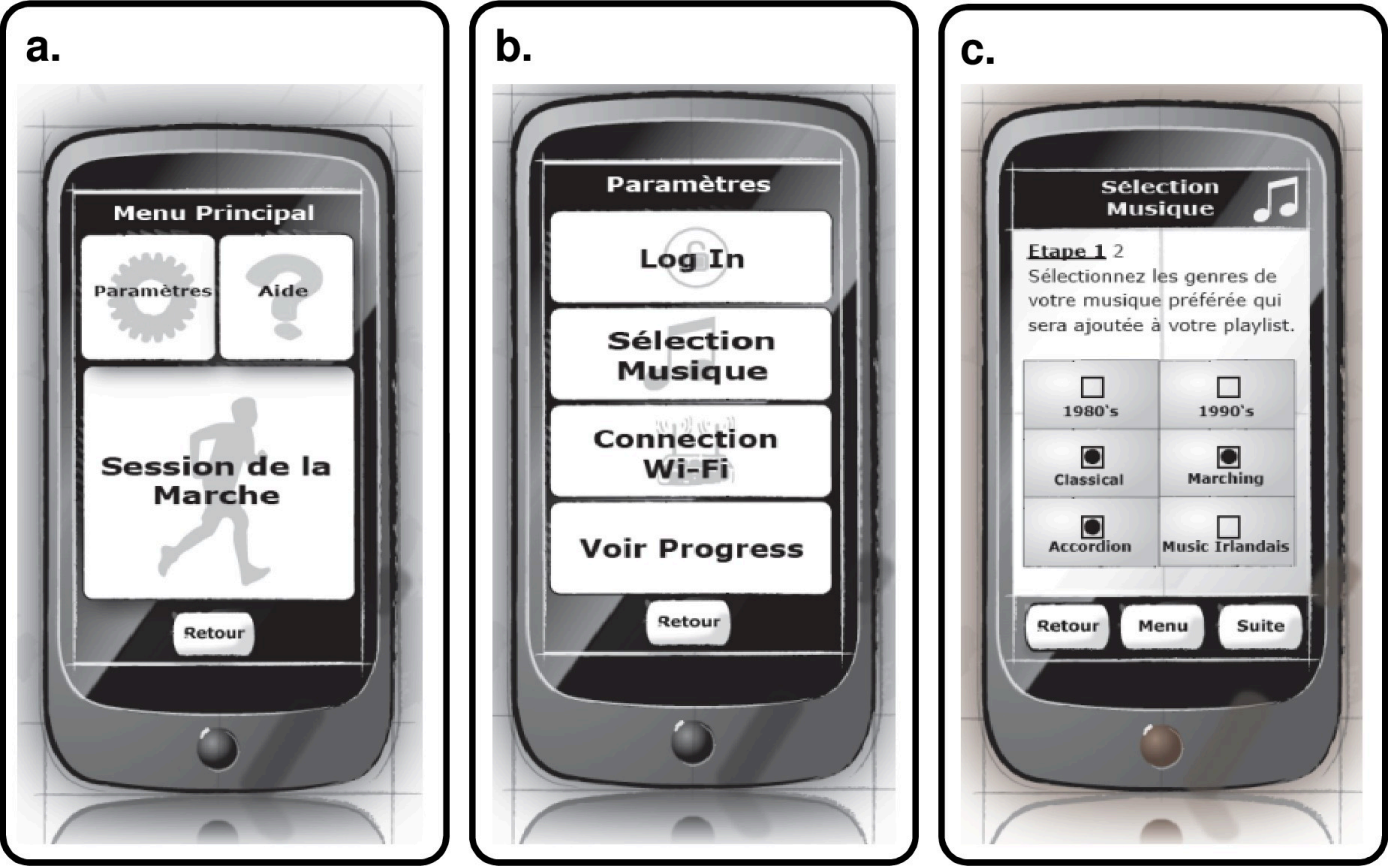
Paper prototype screens used in second iteration. Text is in French as before. (a) Main menu, (b) settings menu, (c) music selection by genre.

As result of the analysis of the first iteration of the mobile application several changes were implemented and some of these can be seen in Fig 8:

- The main menu screen was simplified by reducing from nine buttons to three larger buttons that let users find the main functions easily (Fig 8A).
- Features which are rarely required and are not generally part of the main flow were moved to a settings screen. This included cloud login (usually only required on first use), connecting the WiFi (usually only required when first using the phone at home), and choosing preferred music genres (usually expected to be set up on first use and rarely changed). To simplify the UI, access to the user’s walking program progress information was also placed on this menu (Fig 8B).
- Explanation screens were trimmed because participants commented that those instructions were not needed every time that the application was opened. In the previous iteration all the explanations were presented at the beginning every time.
- The top menu buttons which were present on every screen in the previous version were replaced by a single menu button at the bottom of each screen that always brought the user back to the main screen. This was the only top menu button in the previous version that proved useful to participants.
- To reduce the cognitive load of PwPD using the interface and simplify the appearance, the graphical “breadcrumb trail” used to show progress through a set of related screens was replaced by a text indicating the number of steps and which step was currently active.
- In the music selection screen, the music selection genre “buttons” added a check box to indicate more clearly that multiple genres could be selected (Fig 8C).
- The design of the screen flow used to prepare for a walk, take a walk, and save the results of a walk was substantially overhauled. In the revised flow, preparation and taking a walk are integrated into a single flow. Moreover, in this version of the design there was no explicit option to save the results of a walk as it was intended that the mobile application would now do this automatically.

The second iteration of the mobile application was again evaluated using the Wizard-of-Oz method by four PwPD (two female, two males, 46–84 years old). The same protocol, inclusion, and exclusion criteria were used for this second iteration as had been used with the first iteration.

In this case, the participants were asked to perform five different tasks. The previous iteration’s *connect to WiFi* task was excluded from evaluation because the design of this task had not been changed. Instead, participants were asked if they were familiar with connecting to wireless internet at home and if they would be able to connect to WiFi by themselves or would need someone else’s help. The *prepare for walk* and *complete walk* tasks (number 7 and 9) from the previous iteration were combined into a single task in accordance with the design changes.

The results of the second iteration of the evaluation are presented in Table 2 which shows the tasks that were evaluated and the main results obtained. The metrics were calculated identically to those in the previous iteration. Tasks number 1 and 2 were performed with a real smartphone using the Android OS instead of using the paper prototyping because those two tasks do not require any BeatHealth specific functionality. The remaining tasks were performed using low fidelity paper prototypes as in the previous iteration.

**Table 2:** Task evaluation results for the mobile application–Second iteration.

Tasks		Completion score				Completion score	Error rate	Average time on tasks
		P1	P2	P3	P4			
1	Turn on the phone	3	1	2	1	75%	31%	0:23
2	Open the application	3	1	1	1	75%	19%	0:26
3	Log In	2	1	2	2	100%	25%	3:53
4	Select music	2	2	2	1	100%	13%	1:33
5	Prepare and complete walk	2	2	2	1	100%	0%	4:47

The main findings of this evaluation were:

1. The four PwPD who participated in the evaluation showed great variation in performance level when using a smartphone device (tasks number 1 and 2). Some of the PwPD needed more guidance in order to understand the basic functionality of a smartphone and a touch screen, whereas one PwPD exhibited a high level of autonomy during tasks.
2. In general, all PwPD were able to complete the tasks given if the evaluator provided guidance when needed. The overall opinion was that the application was user-friendly and simple, and that there were sufficient instructions.
3. In terms of manipulating the device, no usability problems were observed and the visual properties of the screens were clear.

Completion rates (evaluated as in mobile first iteration) were high even if slight guidance was still needed (see Table 2). This indicates that the usability of the application was improved according to both the end-users’ comments and comparison to the previous version. Lower error rates were observed in second iteration than in first iteration. In the case of the previous design, the highest error rates were associated with main menu interactions and this was changed for the second iteration (fewer buttons). Due to differences in menu structure, direct comparison of identically named tasks in the first and second iterations should be made with some caution. For example, participants did not always find the structure of the simplified menu intuitive because they did not initially know where to look for some functions such as log-in and choosing the music. Both these functions were now accessed by first choosing settings on the main menu screen and the reason for this design change was that these functions are not frequently needed. Therefore, these functions are not assumed to have much effect on every-day usability of the application. Moreover, participants reported that they found the application easy to learn after practicing.

As explained previously, the Wi-Fi connection task was removed in this iteration, but participants were asked about their ability to do this task. All the participants had wireless internet connection at home and three out of four used internet frequently, whereas two out of four reported that they would need help from a relative to connect their device to internet at home. Imperfect completion rates on task one and task two were the result of problem interactions with the phone hardware and the fundamental application launch feature of the Android operating system respectively. Changing either the hardware or operating system was outside the scope of this work and therefore these tasks would be addressed by additional user training or assistance from a relative where necessary.

The SUS rating for the mobile application second iteration indicated higher user satisfaction (75) than the previous iteration (59). Even with a limited number of participants, this indicates a usability improvement over the previous version. This score can also be converted to a percentile rank of 70% which means that it has higher perceived usability than 70% of all products tested [47].

#### Website evaluation

**Fig 9:**
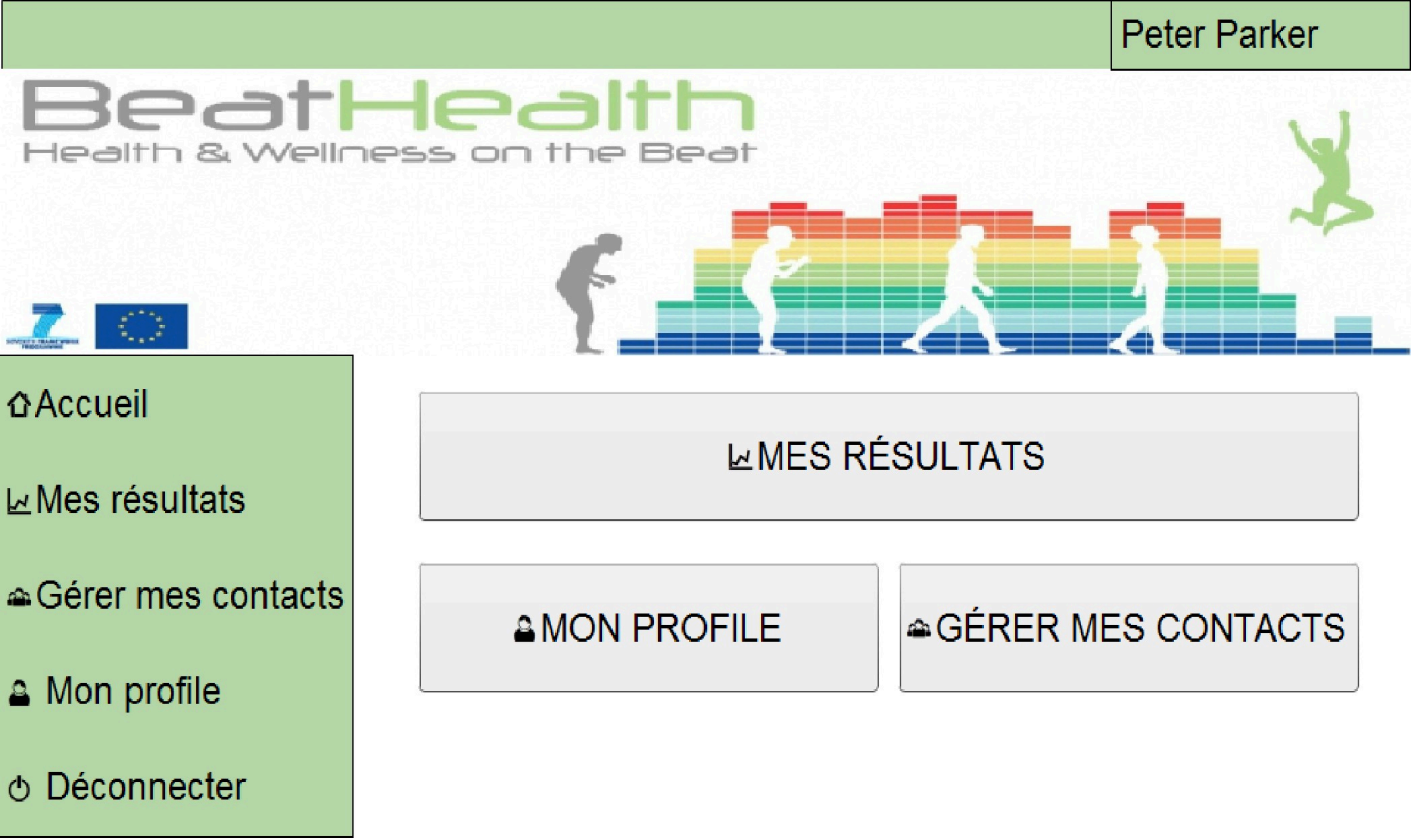
End-user website mock-up. *Main menu* screen in French.

**Fig 10:**
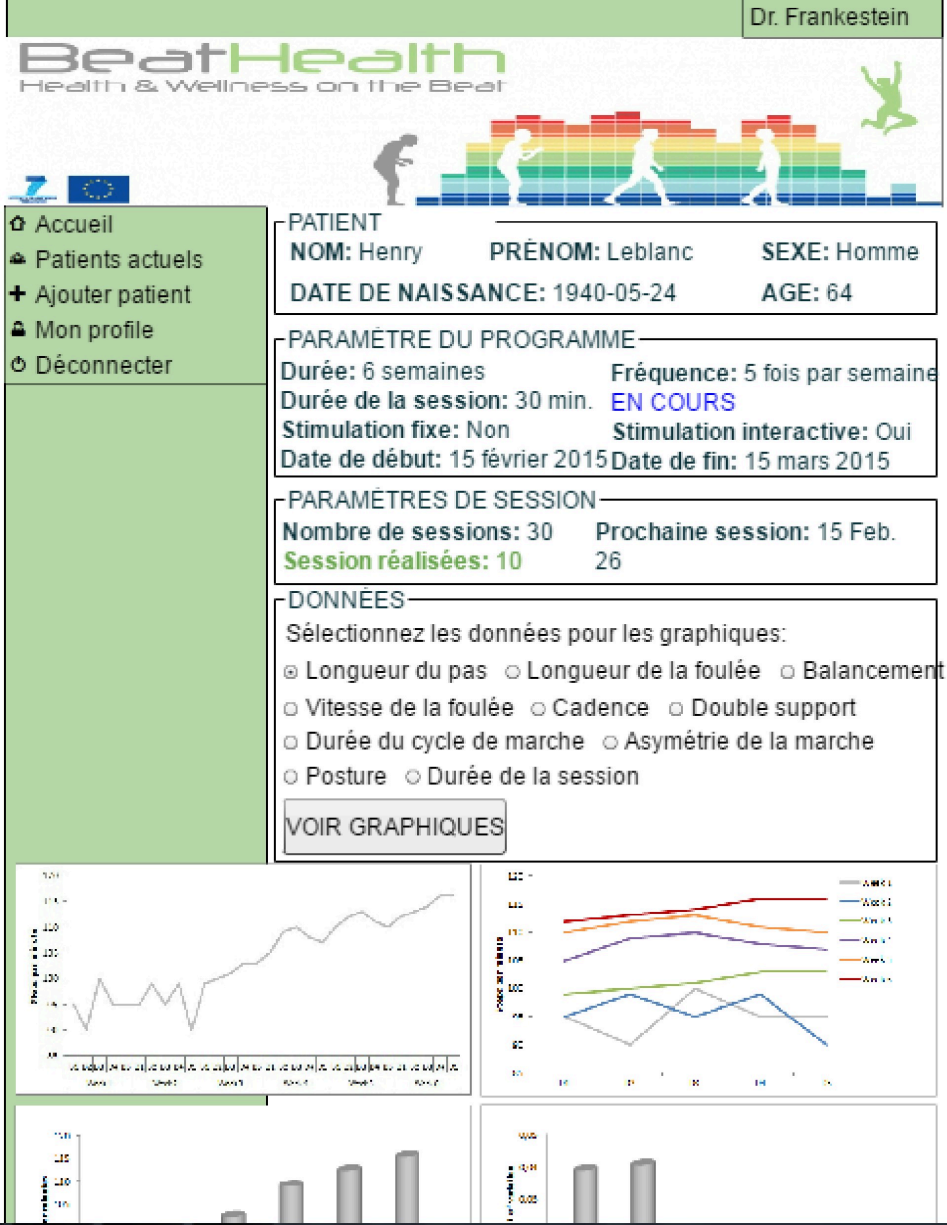
Health professional website mock-up. *View patient results* example in French.

As detailed previously, the purpose of the website in BeatHealth is primarily to provide access to the data that is recorded and summarized from end-user (PwPD) walks using the BeatHealth mobile application. There are different websites functionalities for end-users (PwPD) and health professionals. Fig 9 shows the main menu for end-users as an example while Fig 10 depicts the page used by health professionals to view one patient’s summary results.

The two different user types have access to different functionalities after log-in and therefore, different mock-ups were built for each type of user. The mock-ups were built using FlairBuilder [49] so that they were interactive and could be tested on a personal computer. Real underlying functionality was not implemented at this stage and the aesthetic design was also not a focus at this point. Instead, mock-ups were built in order to validate usability and utility.

The website mockups were evaluated by seven participants representing end-users (six PwPD and one relative) and 11 participants representing health professionals (two neurologists, two rehabilitation doctors, three general practitioners, three physiotherapists, and one medical secretary involved in PwPD care).

The two different mockups (for end-users and for health professionals) were evaluated using different sets of tasks appropriate to their user type and these are detailed in the evaluation results that follow.

All participants (end-users and health professionals) made some comments about problems they encountered while navigating the website. In practice, many of these problems were related to the fact that the evaluated design was a mock-up and did not implement all functionalities that they expected to work. Consequently, these comments there were not related to the utility of the website and they were assigned a lower priority in the evaluation.

Table 3 lists the set of tasks designed for health professionals.

**Table 3:** Tasks performed by health professionals evaluating the website.

Task num.		Task name
1		Register
2		Login
3	3.1	Add a new PwPD
	3.2	Add a new medication
4		Add a training program to an specific PwPD
5	5.1	View the results for a specific PwPD for a specific Program
	5.2	Get graphs/results for Cadence
	5.3	Export data
6		Edit Modopar medication for a specific PwPD and define 200 mg concentration
7		Remove a PwPD from the list because he/she asked me to remove the access
8	8.1	See my profile
	8.2	Edit username
9		Log-out
10		Remove my account

The results of the website evaluation with health professionals indicated that:

1. All participants were able to perform all the tasks presented during the evaluation without usability problems.
2. Health professionals appreciated a simple and user friendly structure that minimized the need to fill in information (for example, pre-filled options where possible).
3. The main difficulties participants encountered were associated with finding where particular functionality was located. Furthermore, to increase the utility and usability of the website, participants requested that there would be access to more of the actions directly from the main page of the website or as options in the side menu.
4. Professionals had different opinions about the need for certain contents or variables for treatment descriptions and the program results summary.

The tasks evaluated with end-users (PwPD and a caregiver) are listed in Table 4 along with the task completion results of that evaluation. Tasks were evaluated in the same manner as for mobile application, except that in this case error rate and average time for each task was not measured.

**Table 4:** End-users evaluation, task completion rate results.

Tasks		Completion Score							Completion score
		P1	P2	P3	P4	P5	P6	P7	
1	Register	2	3	3	1	2	1	2	71%
2	Log In	1	2	2	1	2	1	2	100%
3.1	View “my results”	2	3	3	1	1	1	2	71%
3.2	View graphics and export them to PDF	2	3	4	2	3	1	3	43%
4.1	View “my contacts”	1	2	3	1	2	1	2	100%
4.2	Give/remove permission to/from a contact to see my results	2	3	4	1	2	1	2	71%
5	View “my profile” and edit my username	2	2	3	1	2	1	1	86%
6	Disconnect	2	2	2	1	1	1	1	100%
7	Remove my account	2	3	3	1	1	1	2	71%

Results for the end-user website mockup evaluation indicated that:

1. On average, end-users were able to use the website and perform the requested tasks without guidance or with little guidance. However, high variation was observed among participants in their ability to perform tasks.
2. Two participants (P4 and P6) were clearly more experienced in using a computer than others and this manifested as higher autonomy and better completion rate during tasks.
3. End-users found the overall structure of the website was simple and user-friendly. Most participants found that after practice and guidance the site would be easy to use. Difficulties encountered were mainly due to this being the first time using the website.
4. The main problems encountered were the result of visual properties in the web pages, particularly during tasks 3.1, 3.2, 4.1 and 4.2 in Table 4.
5. Some participants commented that the .*pdf* file format was not familiar (see task 3.2 in Table 4 where results indicate low scores).

Some problems related to the visual characteristics were identified during the design of the website. For example, data graphs were not generated automatically by the website, because it was a mock-up, but were instead just fixed pictures. In addition, the mock-up website was not a responsive design that could scale to the end-user display device and preferences. Consequently, the font size was too small for some end-users. It was decided that these problems would be addressed when implementing the real website rather than by creating another iteration of the website mock-ups.

In general, PwPD encounter some difficulties using a computer because of the effects of PD and, and may have problems using websites as a consequence [50]. Nevertheless, high completion rates were observed among PwPD, and problems mainly occurred when a PwPD could not read the text on the screen. Therefore, close attention should be paid to website responsiveness and visual features during the website development in order to achieve higher usability for PwPD having visual problems.

In summary, the evaluation with health professionals indicated no significant usability problems and indeed that these users positively valued the usability of the website mock-up. Nevertheless, health professionals did make some recommendations to improve the utility and usability of the website by adding more functionality to the main page and/or side menu. PwPD did encounter some usability problems but these were mostly due to visual problems and occasionally problems due to lack of familiarity on first access to the website (which reduced on subsequent accesses). In general, the opinion of the website design was very positive within both user groups.

## Final implementation and evaluation

After a two-year iterative design and implementation process, the final BeatHealth system was implemented. This system comprised custom wearable sensors that measure biomechanical data during gait training, a fully functional mobile application that was connected to the sensors to receive gait data and incorporated the BeatHealth technology and algorithms to perform real-time music adaptation, and two fully functional websites, one for end-users and one for health professionals. The user interface designs of the final mobile application and websites were both based upon the user interface mock-ups that had been evaluated during design and development, but nevertheless included some changes.

There were a number of changes to the user interface design for the final Android mobile application. The two most notable changes were to the home screen and the screen flow associated with taking a walk. The number of buttons on the home screen was increased to five by removing the back button and adding direct access to the music selection and user progress screens. The back button was removed because it is already part of the standard Android user interface. Direct access to music selection was added so that users could more readily change the genres from which songs would be taken for a given session depending on their mood. To promote user engagement and motivation, direct access to user progress was added. In previous evaluation sessions users were especially interested in motivational aspects of the application, so developers considered that giving them direct access to this information would help to engage them. In this version of the design, progress is always shown at the end of a training session, but direct access from the home screen allows a user to easily review their progress to date without starting a training session or just before a training session.

**Fig 11:**
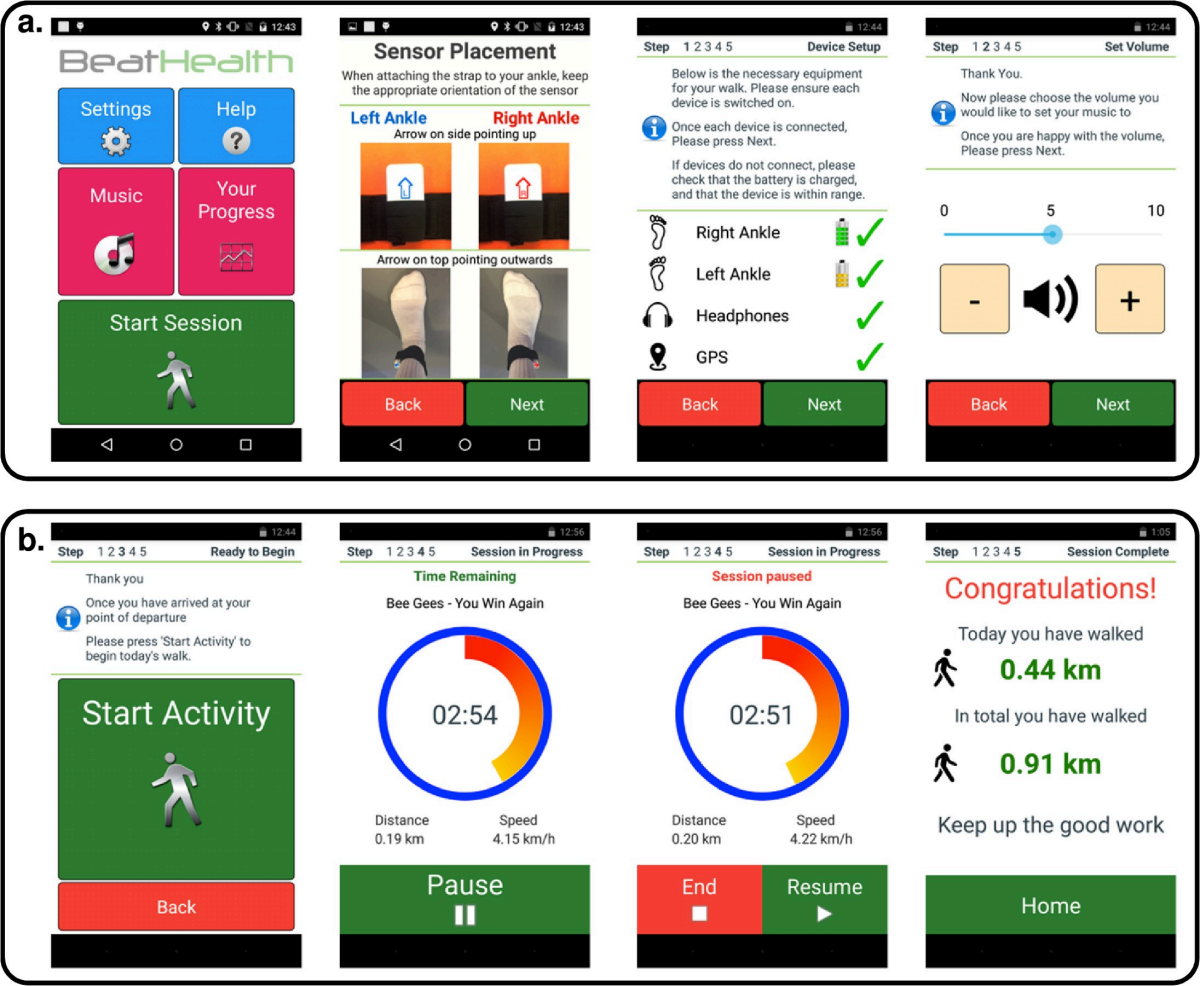
BeatHealth mobile application session screen flow. (a) Screen flow from home screen through preparation for session. (b) Continued screen flow from the end of session preparation, through the session itself and eventually the end of session and progress screen.

The main screen flow associated with a training session was further refined from the final mobile mock-up (second iteration). The flow now includes a screen to help users place the sensors correctly as users had some confusion with this step. (Improved design of the sensor case and attachment mechanism could make correct sensor placement more intuitive, but was outside the scope of this work.) The main session flow is shown in Fig 11.

The button and font sizes were based on recommendations arising from the mock-up evaluations where possible. Nevertheless, minimizing the number of steps or screens in the flow was prioritized with the aim of minimizing the cognitive load. For this reason, some explanatory text, which is useful mainly during early use of the application, was a little smaller than recommended so that it could fit in the space available without requiring additional screens. Although early evaluations had suggested that a tick symbol (used to indicate that good connection or signals had been established) was not sufficiently universal, no better alternative was identified and so it was retained. Color was added so that both symbol and color could be used to recognize the meaning (taking into account that some users may be color blind).

Other modifications relative to the mock-ups included the session in progress screens and the overall training program progress screen (Fig 11B). The session in progress screens used a circular progress bar that also incorporates color coding to provide the user with unambiguous feedback regarding their progress through a given training session. The training plan progress screen was simplified from the concept of graphical progress across a map of France that had been present in the mock-ups. This change was made in order to present clear numeric feedback regarding the total distance covered, to simplify the implementation, and to avoid any negative feedback that could be taken from first designs.

**Fig 12:**
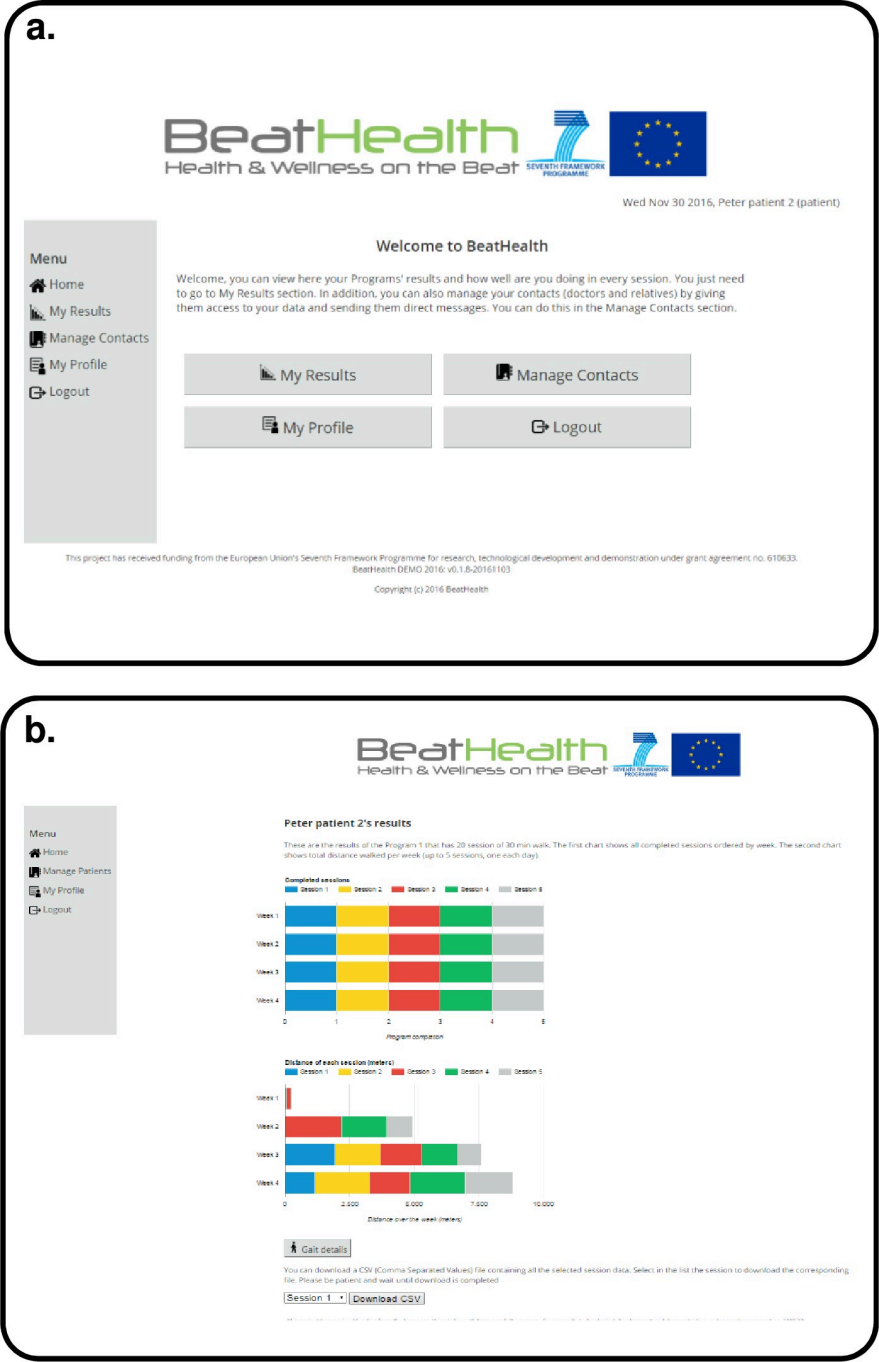
Final design of the BeatHealth website. (a) End-user website example. *Main menu* screen. (b) Health professionals’ website. *Patient results* screen example.

There were a number of changes to the final implementation of the website also. The underlying functionality was implemented such that graphs and data were generated dynamically as needed. The aesthetics of website page design was maintained as much as possible as can be seen in Fig 12 because the presented design had been positively valued by the participants’ evaluation (both by PwPD and health professionals). The major change to the website was to make it a responsive design (that is, font and button sizes change dynamically depending on the computer and browser settings) thereby ensuring the proper font and graph size as demanded by participants during evaluation phase.

Field trials were evaluated and approved by the Ethical Committee *Comité de protection des personnnes Sud Méditérranée III* (France) on the 8^th^ of April 8 of 2015, under protocol number 9193–4. The Protocol was designed according to best clinical practices and Declaration of Helsinki.

The final BeatHealth system was evaluated by 37 PwPD (whose demographic information can be found in S2 Table). Each participant was informed about their participation and personal data treatment, and informed consent was signed when they decided to participate. Participants received brief training on how to use the mobile application and sensors at the hospital and after that they took the system home to use during a three-month period. It was intended that participants would also access the website during the three-month evaluation period. In practice, there were problems saving data from trials to the cloud early in the evaluation period. These problems were solved during trials but the consequence was that there was much less use of the website by participants than intended. For this reason no data about the usability and utility of the final website could be recorded. As participants completed the field trial they were asked to fill in two questionnaires, one of which was an SUS validated questionnaire [46, 47]. A copy of the questionnaires can be found in S4 File. The questionnaires were presented in French because the participants were French speakers, so part of the questionnaire is directly reproduced in this language. This questionnaire was generated to ask to participants about their perceptions at the end of their participation. One of the participants did not complete de questionnaire. Some of the results from the remaining participants are listed in Table 5:

**Table 5:** Questionnaire results.

Lang	Question	Results Number (Percentage)		
		Agree	Neutral	Disagree
F	Globalement, je suis satisfait(e) de la facilité avec laquelle on utilise BeatHealth.	28 (78%)	5 (14%)	3 (8%)
E	Overall, I am satisfied how ease can be BeatHealth used.			
F	C’était facile d’apprendre à utiliser BeatHealth.	26 (72%)	5 (14%)	5 (14%)
E	It was easy to learn how to use BeatHealth.			
F	Chaque fois que j’ai fait une erreur avec BeatHealth, j’ai pu la corriger facilement et rapidement.	14 (39%)	13 (36%)	9 (25%)
E	Whenever I made a mistake with BeatHealth, I was able to fix it easily and quickly.			
F	Les informations données par BeatHealth étaient faciles à comprendre.	26 (72%)	7 (20%)	3 (8%)
E	The information given by BeatHealth was easy to understand.			
F	La présentation sur l’écran était claire.	31 (86%)	3 (8%)	2 (6%)
E	The presentation on the screen was clear.			

Lang, language could be French (F) or English (E). Questions are represented in French as they were designed and used, and in English, translated for the publication.

The results show the number of participants and the corresponding percentage in parentheses for each answer.

The results indicate that in general participants were satisfied with the application (78%), it was easy to learn (72%), the information provided by the system was easy to understand (72%) and clear enough (86%). On the other hand, most participants encountered difficulties recovering from problems or mistakes (61%).

The most commonly encountered problem with the system was related to physical components such as the phone buttons or sensors (seven participants), but some participants also reported problems with the application (four), cloud connection (two), and GPS (two).

The average SUS rating for the 37 participants was 78.65 which was slightly higher than the mock-ups evaluated previously. In this case, participants used the system for longer, and could rate not only the application interface, but also the functionality and different components (including for example, sensors and headphones) of the BeatHealth system. The 78.65 SUS rating converts to a percentile rank of 70% using the method of Sauro [48]. This is the same percentile rank as the mock-up evaluated previous and the SUS rating would need to increase somewhat to 80.3 in order to convert to the next percentile rank of 90% (the top 10% of scores) which is also the point at which users are more likely to recommend the product to a friend.

PwPD were asked about what they would change, add, or remove if they could. Most of the responses were related to physical components or to the choice of music and not to the application itself. Among the participants, 12 reported that the BeatHealth system helped them with their gait or made them walk more, while five participants indicated that BeatHealth had not helped them.

## Discussion

The BeatHealth system was developed based on the RAS technique to improve gait and reduce freezing episodes on PwPD. During the project design and development field trials were conducted to evaluate the effectiveness of the system. In this study, the subjective perception of the PwPD who used the system for three months prescribed by their physicians has been analyzed. Results show that only 32% of participants subjectively felt that the system helped them improve their gait. Additional understanding of this subjective result could be obtained by comparison with objective measurements of gait change during the trials. Such objective measurements require detailed processing and analysis of the recorded sensor and GPS data but this processing and analysis is outside the scope of the work presented in this paper.

The co-design methodology and tools selected in the project enabled the involvement of a number of stakeholders including PwPD, caregivers, and health professionals, from the very beginning. The use of low fidelity prototypes and mock-ups enabled the project team to build prototypes rapidly and inexpensively. This facilitated an iterative design process because more designs could be created and evaluated with end-users in a shorter time allowing more opportunities to refine the designs.

The final evaluation of the system showed it was usable, but also indicated areas for improvement. Despite most of the participants expressing their satisfaction with the application, and stating that the system and the information it presented was easy to understand and clear enough, 61% of participants had difficulties recovering from errors, indicating there are still problems in the system which must be identified and solved. The problem areas must also be better explained to the final users and they must be given better tools to recover by themselves. Giving hints or additional information to final users can help them recover from identified problems, particularly if they are not familiar with new technologies. Helping users recover from mistakes could significantly improve the usability of the system.

The role of the family of PwPD and health professionals has also been considered from the beginning of the project as they were considered important for the BeatHealth system design and development. During the project development we realized that informal caregivers needed an application that could be used by PwPD independently. Health professionals in this case have a very important role, because the BeatHealth application may be used to improve gait and reduce freezing episodes. Sessions must be controlled and customized for each user, and the physician must be responsible for that. The participation of health professionals in BeatHealth design process was crucial.

Recruitment of PwPD and health professionals proved to be challenging and for this reason the number of participants was not very high in some cases, especially in the preliminary phases. The main issue recruiting health professional participants was their workload. Nevertheless, we considered that the number of participants is sufficient for these evaluations in accordance with Nielsen [51].

There are some limitations to the study. The acceptance of the system was not measured. It is not only the usability of a system that is important, but also the acceptance of it, particularly if it is expected to be used on a continuous and daily basis. Adherence was also not evaluated in this study. During the design process, PwPD suggested adding motivational aspects to use the system. It may be possible to use the BeatHealth system as a treatment to improve gait for PwPD and it would therefore be important to measure adherence and engagement. In the field trials, engagement was difficult to measure because participants only used the system for three months. In the future, adherence should be evaluated over longer trials in order to check the importance of motivational aspects related to treatments benefits.

One final limitation of the study is the limited data regarding the website. Due to their limited availability, physicians evaluated the website prototype only once. Moreover, they did not use the website during the field trials to monitor their patients’ sessions. The PwPD, in turn, did not use the website much due to a cloud data synchronization problem noted previously and because the mobile phone application was convenient and appeared to provide sufficient information for them.

## Conclusions

In the BeatHealth project, it was important to involving different stakeholders (health professionals, informal caregivers, relatives and PwPD) from the very beginning of the project. The aim of this approach was to produce the most useful, usable and easy-to-use final system possible, with the most appropriate functionalities. To achieve this, different tools and techniques such as interviews, personas, use cases, ethnographic observation, interviews, paper prototyping, mock-ups, usability evaluation and field-trials were used. These tools gathered important information needed to generate the first version of the application and subsequently improve the design. The BeatHealth system is formed by three applications: a mobile phone application, and two websites, one for PwPD and the other for health professionals. For both mobile application and websites, some literature guidelines were taken into account when building the first mock-up versions, but, in the case of PwPD, recommendations are difficult to find, particularly for websites. Evaluations of the final versions of both the mobile application and websites yielded good results. These results were assessed though a validated questionnaire (SUS questionnaire), so we can conclude that a complete methodology that involved stakeholders from the very beginning until the final evaluation of the design proved effective at producing a usable system in this particular case.

## Supporting information

S1 File Functional requirements for the BeatHealth mobile application. (DOCX)

S2 File Functional requirements for the BeatHealth website for the end-users (PwPD). (DOCX)

S3 File Functional requirements for the BeatHealth website for the health professionals. (DOCX)

S4 File Questionnaires used in BeatHealth Evaluation Phase. (PDF)

S1 Table Use Case template. (DOCX)

S2 Table Demographic data of participants in field trials. (DOCX)

S1 Dataset Participants’ data regarding BeatHealth evaluation. (XLSX)
