# Supplementary material for: Design and development of a gait training system for Parkinson’s disease
Source: PLoS One. 2018 Nov 12;13(11):e0207136. doi: 10.1371/journal.pone.0207136 (PMC6231661; doi:10.1371/journal.pone.0207136)
Supplement: S1 File — (DOCX) [file pone.0207136.s001.docx]

Functional requirements for the BeatHealth mobile application:

1. The application should guide the participants through each stage of the process to improve the probability of successful use by novice users.
2. The application should offer the option of skipping the guides to more advanced participants if they are not required.
3. A user name and password is needed for data security reasons. User should log into the application to ensure that personal information is safe in the application. User will have the possibility of storing this information on the phone on the very first access to automatically access to the application without explicit log-in every time.
4. The application must be capable of identifying if the BeatHealth movement sensors and headphones are connected and/or paired. Once they have configured this information it will be stored locally to avoid the need of choosing the sensors every time the application is used.
5. The application must be capable of reading the information from the sensors. Information like sensor connection strength and battery level should be also read and provided to the user so that they may understand the status of the sensors, particularly if a problem arises.
6. Gait data will be processed locally by the mobile application and sensors with the aim of being temporarily stored in a private folder in the phone. This will be more transparent for users whilst maintaining privacy.
7. The application must be capable of reading music from the device. The application must look for music in the smartphone memory and let the user select a playlist or individual songs.
8. The application should generate a personal code for the user to delegate access to health professionals to their personal data through the website.
9. When started, the application should automatically select the appropriate new session already specified by the health professional via the website.
10. The user must have the option of pausing or stopping the session (and hence the music) and being able to resume the session shortly afterwards when required.
11. When session is stopped or finished the user must see a summary of the results of his/her session. At the same time, all the registered information must be automatically remotely saved and removed from the local system.
12. When the session is stopped user must have the option to change configuration: add/change the sensors, change selected music, set the volume, generate a new code for access delegation, or log-out.
13. All the normal functionalities of a smartphone should be active in background (for example, receiving calls or messages). When a call is received the system should pause the session to allow talking by phone and start over again when the call is finished.
14. The application must have a social and motivational element to increase the potential of the successful completion of the training.
