# Supplementary material for: Design and development of a gait training system for Parkinson’s disease
Source: PLoS One. 2018 Nov 12;13(11):e0207136. doi: 10.1371/journal.pone.0207136 (PMC6231661; doi:10.1371/journal.pone.0207136)
Supplement: S2 File — (DOCX) [file pone.0207136.s002.docx]

Functional requirements for the BeatHealth website for the end-users (PwPD):

1. A user name and password is needed for security reasons. User should log into the website to ensure that personal information is safe. The system must remind the user to change password every six months to fulfill the security measures of data protection Directive [1] which was applicable when BeatHealth website was designed.
2. The user can introduce his/her data to create a new account, but it only can be created from the website.
3. The health data stored should be saved with special security measures because it is considered especially sensitive.
4. The application must show the user the session and summary results, preferably in graphical form.
5. It should be possible to download results as reports in CSV or PDF files.
6. The user profile must be managed in the website: the user may modify his/her data or remove his/her account. In case that remove the account all the information associated must be also completely removed from the system.
7. The user should have the opportunity to manage the access delegations to his/her data from this website, in particular, removing delegation or creating new codes to give authorization to third parties (i.e. health professionals).
8. The user could log-out from the website. Automatic log-out should occur if there is no activity for more than three minutes due to security issues.
9. Haensch K, Atienza Serna L. Directive 95/46/EC of the European Parliament and of the Council of 24 October 1995 on the protection of individuals with regard to the processing of personal data and on the free movement of such data. Luxemburg: European Parliament and of the Council; 1995 Oct. Official Journal L 281, 23/11/1995 P. 0031 - 0050
