# Supplementary material for: Design and development of a gait training system for Parkinson’s disease
Source: PLoS One. 2018 Nov 12;13(11):e0207136. doi: 10.1371/journal.pone.0207136 (PMC6231661; doi:10.1371/journal.pone.0207136)
Supplement: S3 File — (DOCX) [file pone.0207136.s003.docx]

Functional requirements for the BeatHealth website for the health professionals:

1. A user name and password is needed for security reasons. Health professional should log-in to the website to ensure that the PwPD’s personal information is safe. The system must remind the user to change password every six months.
2. New user could provide his/her personal data to create a new health professional account, but new accounts must be created only from the website.
3. A health professional must not have access to any PwPD’s personal information unless the PwPD has explicitly granted the access. Health professional must have the option of adding a new PwPD to the patient list with the access delegation code that the PwPD will facilitate to him/her.
4. The data retrieved from a PwPD must not be stored in any other database but only shown on the screen. Access to PwPD’s data should be done through secure channels.
5. Once that a PwPD grant access to a health professional, this person could view, edit, add, or remove the following data related to the PwPD:
   1. Demographic data: age, weight (only view).
   2. Treatment and medication management (edit/add/remove).
   3. Programs and session management (edit/add/remove).
   4. Program results, only view on screen or download this information into CSV or PDF documents options for medical proposals. Health professionals asked for the option of converting patients’ data into files because they find easier managing data in those formats. But according to their Hippocratic Oath and their compromise with patients they will only use those data into the clinic and for medical purpose.
6. The health professional must be able to remove a PwPD from his/her patients list.
7. The health professional’s profile must be managed via the website. The user may modify his/her personal data and/or may remove his/her account. In case that they decide to remove the account all the information associated with that user must also be completely removed from the system.
8. The health professional could log-out from the website. Log-out will automatically occur if there is no activity for more than three minutes due to security issues.
