# Supplementary material for: Design and development of a gait training system for Parkinson’s disease
Source: PLoS One. 2018 Nov 12;13(11):e0207136. doi: 10.1371/journal.pone.0207136 (PMC6231661; doi:10.1371/journal.pone.0207136)
Supplement: S4 File — (PDF) [file pone.0207136.s004.pdf]

Questionnaire for Patients

Participant Code______

1. Gender : ☐ Male ☐ Female
2. Could you tell us your age range?

< 65	65-69	70-74	75-79	80-84	85+

3. Do you own computer or laptop? ☐ Yes ☐ No
4. How often do you use your computer?

Every day	2-6 times a week	Once a week	Less

5. Do you own a mobile phone? ☐ Yes ☐ No

   If you do, is it a smartphone (with touch screen)? ☐ Yes ☐ No

Questions about the system

6. Did you find especially difficult to perform any action? ☐ Yes ☐ No

   If you did, please, list them.

   ____

   ____

   ____

7. Did you find especially difficult access any link/button? ☐ Yes ☐ No

   If you did, please, list them.

   ____

   ____

   ____

8. Did you find difficult reading texts? ☐ Yes ☐ No

   If you did, please, list them.

Questions about the smartphone app

9. Did you need help from someone to use the BeatPark app? ☐ Yes ☐ No
10. Would you recommend BeatPark app to your friends/other Parkinson patients?
    ☐ Yes ☐ No

If you **DO NOT;** could you explain us why?

SUS questionnaire for usability about BeatPark app in the smartphone

	1 (strongly disagree)	2	3	4	5 (strongly agree)
11. I think that I would like to use BeatPark app frequently.					
12. I found BeatPark app unnecessarily complex					
13. I thought BeatPark app was easy to use					
14. I think that I would need the support of a technical person to be able to use BeatPark app					
15. I found the various functions in BeatPark app were well integrated					
16. I thought there was too much inconsistency in BeatPark app					
17. I would imagine that most people would learn to use BeatPark app very quickly					
18. I found BeatPark app very cumbersome to use					
19. I felt very confident using BeatPark app					
20. I needed to learn a lot of things before I could get going with BeatPark app					

This part is only for patients who used the web-site. If you DID NOT, go to question num. 36.

21. How often did you use the web-site during the trials?

Every day	2-6 times a week	Once a week	Less

Other general questions

22. Would you add something to the system?

23. Would you remove something to the system?

24. Would you change something to the system?

25. Any additional comment?

	Pas d'accord	Neutre	D'accord
Je pense que j'aimerais utiliser BeatHealth souvent.			
J'ai trouvé BeatHealth très compliqué.			
J'ai trouvé que BeatHealth était facile à utiliser.			
Je pense que j'aurais besoin du support technique d'une personne pour être capable d'utiliser BeatHealth.			
J'ai trouvé que les différentes parties de BeatHealth étaient cohérentes entre elles.			
J'ai trouvé que les différentes parties de BeatHealth n'étaient pas très cohérentes entre elles.			
J'imagine que la plupart des gens pourrait apprendre à utiliser BeatHealth rapidement.			
J'ai trouvé BeatHealth très difficile à utiliser.			
Je me suis senti(e) en confiance en utilisant BeatHealth.			
J'ai dû apprendre beaucoup de choses avant de pouvoir utiliser BeatHealth.			
Globalement, je suis satisfait(e) de la facilité avec laquelle on utilise BeatHealth.			
J'ai été capable de passer rapidement d'une étape à l'autre du programme BeatHealth.			
J'ai trouvé BeatHealth ergonomique.			
C'était facile d'apprendre à utiliser BeatHealth.			
Chaque fois que j'ai fait une erreur avec BeatHealth, j'ai pu la corriger facilement et rapidement.			
Il a été facile de trouver les informations dont j'avais besoin.			
Les informations données par BeatHealth étaient faciles à comprendre.			
La présentation sur l'écran était claire.			
Si j'ai accès à BeatHealth, je l'utiliserais.			
Je suis satisfait(e) de BeatHealth.			
Je recommanderai BeatHealth à un ami.			
BeatHealth est amusant à utiliser.			
BeatHealth fonctionne comme je le veux.			
Je sens que j'ai besoin de BeatHealth.			
BeatHealth m'a aidé à contrôler mes symptômes.			
BeatHealth était assez interactif.			

From Ben-Zeev D, Brenner CJ, Begale M, Duffecy J, Mohr DC, Mueser KT. Feasibility, acceptability, and preliminary efficacy of a smartphone intervention for schizophrenia. Schizophr Bull. 2014 ;40:1244-53
