# Supplementary material for: Design and development of a gait training system for Parkinson’s disease
Source: PLoS One. 2018 Nov 12;13(11):e0207136. doi: 10.1371/journal.pone.0207136 (PMC6231661; doi:10.1371/journal.pone.0207136)
Supplement: S1 Table — (DOCX) [file pone.0207136.s005.docx]

| **Variables** | **Number** | **Percentage** |
| --- | --- | --- |
| **Gender** |  |  |
| Male | 22 | 59% |
| Female | 15 | 41% |
| **Age range** |  |  |
| <65 | 24 | 65% |
| 65-69 | 4 | 11% |
| 70-74 | 3 | 8% |
| 75-79 | 5 | 13% |
| >80 | 1 | 3% |
| **Computer at home** |  |  |
| Yes - Using it every day | 22 | 59% |
| Yes – Using it 2-6 times/week | 5 | 14% |
| Yes – Using it 1 time/week | 2 | 5% |
| No | 8 | 22% |
| **Phone owners** |  |  |
| Smartphone | 21 | 57% |
| Other mobile pone | 16 | 43% |
