# Supplementary material for: Design and development of a gait training system for Parkinson’s disease
Source: PLoS One. 2018 Nov 12;13(11):e0207136. doi: 10.1371/journal.pone.0207136 (PMC6231661; doi:10.1371/journal.pone.0207136)
Supplement: S2 Table — (DOCX) [file pone.0207136.s006.docx]

| **Element name** | **Description** |
| --- | --- |
| Title | One-line description of the Use Case. |
| Description | Short description of the Use Case to explain *user type* (who) who *do some action* (what) and the *desired result* (why) he/she wants. |
| Acceptance Criteria | Acceptance criteria must define the specific details required for the Use Case to be considered complete. Acceptance Criteria are supposed to be true when the use case is marked as done. |
| UI Flow | Graphical representation (an image) of the sequence of screens or states the User Interface (UI) passes through. |
| Comments | Additional useful information affecting the use case from the consortium members. Comments must contain author name and date added, for this reason it is better to use text editor comment feature rather than adding the comment directly into the use case. |
| Relevance | MoSCoW scale. |
| Implemented | Yes/No |
